# Deoxynivalenol Affects Proliferation and Expression of Activation-Related Molecules in Major Porcine T-Cell Subsets

**DOI:** 10.3390/toxins11110644

**Published:** 2019-11-05

**Authors:** Eleni Vatzia, Alix Pierron, Armin Saalmüller, Elisabeth Mayer, Wilhelm Gerner

**Affiliations:** 1 Institute of Immunology, Department of Pathobiology, University of Veterinary Medicine Vienna, 1210 Vienna, Austria; eleni.vatzia@vetmeduni.ac.at (E.V.); or armin.saalmueller@vetmeduni.ac.at (A.S.); 2 BIOMIN Research Center, Technopark 1, 3430 Tulln, Austria; e.mayer@biomin.net

**Keywords:** deoxynivalenol, in vitro, pig, T cells, proliferation, costimulatory molecules, CD27, CD28

## Abstract

The *Fusarium* mycotoxin deoxynivalenol (DON) contaminates animal feed worldwide. In vivo, DON modifies the cellular protein synthesis, thereby also affecting the immune system. However, the functional consequences of this are still ill-defined. In this study, peripheral blood mononuclear cells from healthy pigs were incubated with different DON concentrations in the presence of Concanavalin A (ConA), a plant-derived polyclonal T-cell stimulant. T-cell subsets were investigated for proliferation and expression of CD8α, CD27, and CD28, which are involved in activation and costimulation of porcine T cells. A clear decrease in proliferation of all ConA-stimulated major T-cell subsets (CD4^+^, CD8^+^, and γδ T cells) was observed in DON concentrations higher than 0.4 µM. This applied in particular to naïve CD4^+^ and CD8^+^ T cells. From 0.8 μM onwards, DON induced a reduction of CD8α (CD4^+^) and CD27 expression (CD4^+^ and CD8^+^ T cells). CD28 expression was diminished in CD4^+^ and CD8^+^ T cells at a concentration of 1.6 µM DON. None of these effects were observed with the DON-derivative deepoxy-deoxynivalenol (DOM-1) at 16 µM. These results indicate that DON reduces T-cell proliferation and the expression of molecules involved in T-cell activation, providing a molecular basis for some of the described immunosuppressive effects of DON.

## 1. Introduction

The *Fusarium* mycotoxin deoxynivalenol (DON) is a secondary fungal metabolite, which contaminates cereal-based foods worldwide and, thus, is considered as a threat for public health and also for the nutrition of farm animals [1,2,3]. Pigs are highly exposed to DON and other mycotoxins due to their cereal-rich diet, leading to health problems and impairment of performance traits [4,5]. At a cellular level, DON induces a ribotoxic stress response via binding to the peptidyl transferase center of the 60S unit of the ribosome, which results in elongation inhibition [6,7]. The binding to the ribosome leads to the activation of mitogen-activated protein kinases (MAPKs), which are involved in processes such as cell growth, proliferation, and apoptosis. In vitro experiments on cell lines derived from murine macrophages and human monocytes have shown that this could result in either immunostimulatory or immunosuppressive effects [8,9,10].

For cells of the adaptive immune system, i.e., B and T cells, proliferation is essential for the activation and differentiation of cells into effector and memory subsets. Previous studies have shown that DON impairs cell proliferation, including cells of the immune system [11,12,13]. In the study by Novak et al. [13], the influence of different DON and deepoxy-deoxynivalenol (DOM-1) concentrations on the proliferation of Concanavalin A (ConA)-stimulated bovine, porcine, and chicken peripheral blood mononuclear cells (PBMCs) was investigated. For bovine PBMCs, which showed the highest sensitivity to DON-induced impairment of proliferation, the phenotype of major T-cell subsets was also investigated in combination with proliferation. CD4^+^, CD8^+^, and γδ T cells all showed a similar reduction in proliferation from DON concentrations of 0.4 µM onwards.

In the present study, we extended these analyses to porcine T cells. In addition to studying their proliferation, we also investigated the expression of molecules involved in T-cell costimulation and survival. In particular, CD27 and CD28 have been intensively studied in this context. CD27 is a costimulatory molecule that belongs to the tumor necrosis factor receptor super family. It promotes the survival of activated T cells, even in the absence of CD28 [14]. Studies in mice have shown that it also supports the accumulation of antigen-specific T cells at the site of infection during influenza infection [14,15,16]. It has also been proposed that CD27 signaling contributes to the establishment of a T_H_1 differentiation [17,18]. In accordance with data for murine and human T cells, it has been shown that CD27 is expressed in naïve CD4^+^ and CD8^+^ T cells in the pig. Moreover, triggering of CD27 by specific monoclonal antibodies supports porcine T-cell proliferation [19]. Next to CD27, CD28 is a cell surface marker receptor that belongs to the immunoglobulin superfamily and is essential for T-cell activation, proliferation, and survival [20,21]. CD28 promotes T-cell survival by promoting the expression of the antiapoptotic protein Bcl-X_L [22,23]_. Upon CD28 stimulation, high levels of IL-2 are produced, which enhance the viability and proliferation of T cells [22,24]. In pigs, recently, the development of a CD28-specific monoclonal antibody allowed for investigations that confirmed that in this species CD28 is also expressed by naïve T cells and that CD28 triggering provides a costimulatory signal for proliferation (data currently unpublished).

Next to CD27 and CD28, numerous porcine T cells do express CD8αα homodimers. For porcine CD4^+^ T cells, CD8α has been described as an activation marker, which is upregulated after in vitro stimulation [25]. In combination with other differentiation markers, CD8α has been used to describe the differentiation stage of CD4^+^ T cells [26,27]. Somewhat similarly, the frequency of CD8α-expressing γδ T cells increases in pigs with age [28], suggesting that expression of this molecule is involved in the differentiation of porcine γδ T cells.

Hence, in the present study, we studied in detail the effect of DON on the proliferation of CD4^+^, CD8^+^, and γδ T cells. Moreover, the expression of CD8α, CD27, and/or CD28 was addressed within those T-cell subsets. In addition to DON, we also studied the influence of its derivative deepoxy-deoxynivalenol (DOM-1), which is a microbial biotransformation product of DON with a reduced toxicity [29,30,31].

## 2. Results

### 2.1. Cell Viability and Lymphocyte Proliferation

**Figure 1:**
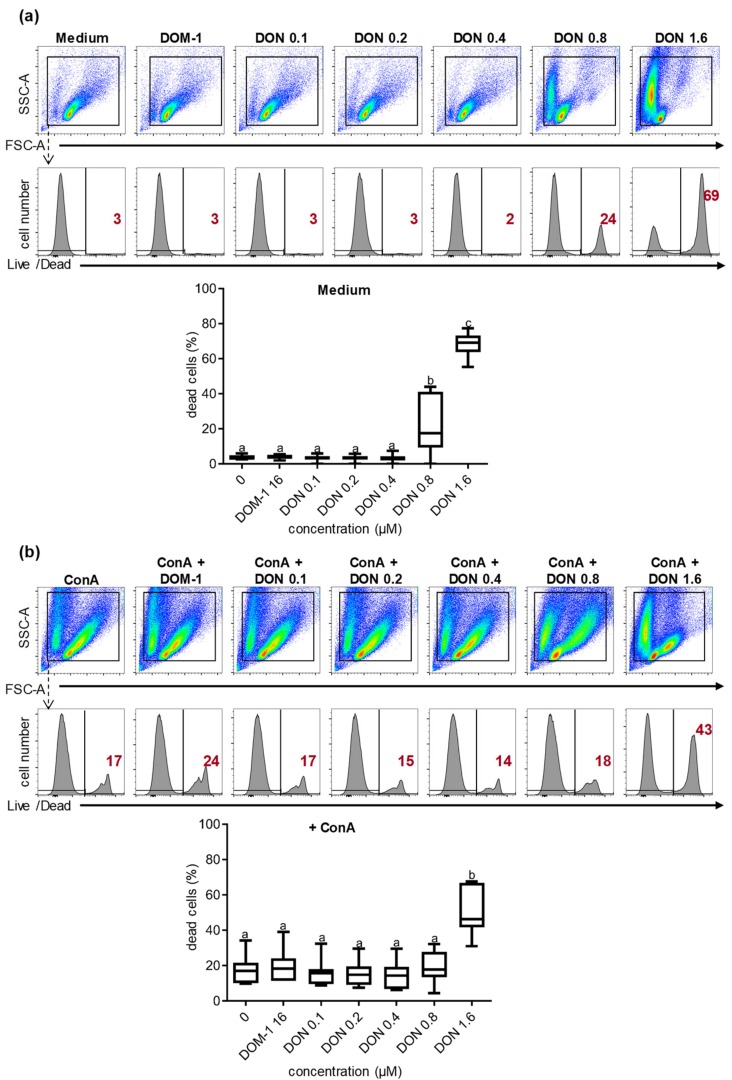
Viability of porcine peripheral blood mononuclear cells (PBMCs) in the presence of deoxynivalenol (DON) and deepoxy-deoxynivalenol (DOM-1). PBMCs were cultivated four days in the absence (**a**) or presence (**b**) of ConA in combination with different concentrations of DON (0.1, 0.2, 0.4, 0.8, 1.6 μM), a single DOM-1 concentration (16 μM, designated as DOM-1), or no DON (designated as medium in (a) or ConA in (b)). Harvested PBMCs were subjected to flow cytometry, gated according to light scatter properties (pseudocolor plots), and analyzed in histograms for fluorescence intensity of a live/dead dye. The solid vertical lines in the histograms separate dead cells on the right and live cells on the left. The numbers located on the right side in the histograms indicate the percentage of the dead cells. Flow cytometry graphs show representative data from one animal out of seven. Different letters on boxplots indicate significant differences. (a) a–b: *p* < 0.001, a–c: *p* < 0.0001, b–c: *p* < 0.0001. (b) a–b: *p* < 0.0001.

Next to proliferation, various in vitro studies conducted in the past have shown that DON can impair the survival of different cell types [32,33]; therefore in this study we initially investigated the impact of DON and DOM-1 on porcine PBMCs viability. PBMCs cultivated either in medium (Figure 1a) or ConA (Figure 1b), together with different DON (0.1–1.6 µM) and DOM-1 (16 µM) concentrations, were investigated. For DOM-1, this 10-fold higher concentration of the maximum concentration of DON (i.e., 1.6 µM) was chosen based on pilot experiments showing that a DON concentration of 1.6 µM completely abolished ConA-induced proliferation (see below).

Data obtained from PBMCs of seven different animals showed that neither 16 μM DOM-1 nor DON concentrations up to 0.4 μM affected the viability of the cells cultivated in medium or ConA. However, at 0.8 μM DON for cells cultivated in medium, a significant increase (*p* < 0.001) of dead cells was observed, which was even higher at 1.6 μM DON (*p* < 0.0001). Similarly, in ConA-stimulated cells (Figure 1b), a significant increase (*p* < 0.0001) of dead cells was observed at 1.6 μM DON but not in lower DON concentrations.

**Figure 2:**
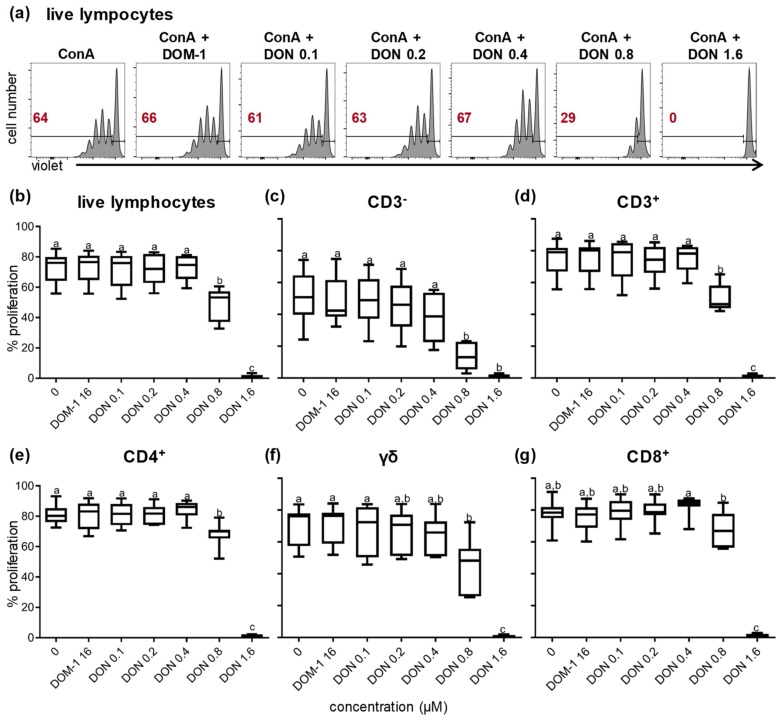
Proliferation of lymphocytes and major T-cell subsets in the presence of DON and DOM-1. PBMCs were stained with violet proliferation dye. After four days of in vitro cultivation in the presence of ConA alone or in combination with various DON concentrations (0.1, 0.2, 0.4, 0.8, 1.6 μM) and a single DOM-1 concentration (16 μM, designated as DOM-1), they were harvested and labeled for expression of the cell-surface markers CD3, CD4, CD8, and T-cell receptor (TCR)-γδ. Lymphocytes were gated by light scatter properties and further subgated for exclusion of dead cells with a live/ dead discrimination dye (Supplementary Figure S1). (**a**) Histograms show proliferation of live lymphocytes under different cultivation conditions (ConA only, DOM-1, DON in different concentrations). The solid horizontal lines separate the parental population (on the right) and the proliferating generations (on the left). The numbers located on the left side in the histograms display the percentage of proliferating cells. Representative data of lymphocytes from one animal are shown. Live lymphocytes (**b**), CD3^−^ lymphocytes (**c**), CD3^+^ T cells (**d**), CD4^+^ T cells (**e**), γδ T cells (**f**), and CD8^+^ T cells (**g**) were gated (see Supplementary Figure S1) and analyzed for proliferation. The boxplots show the percentage of proliferating cells for the different DON and DOM-1 conditions for seven animals. Different letters on boxplots indicate significant differences: (b,d) a–b: *p* < 0.01, a–c: *p* < 0.0001, b–c: *p* < 0.0001. (c) a–b: *p* < 0.05. (e–g) a–b: *p*<0.05, a–c: *p* < 0.0001, b–c: *p* < 0.0001.

ConA is a plant-derived mitogen, which stimulates T cells and provides the ability to study their proliferation. Previous studies have shown that DON can inhibit lymphocyte proliferation [11,12,13,34,35]. However, whether such an inhibition applies to all T-cells or to particular subsets has only been investigated for bovine T cells so far [13]. Hence, live lymphocytes, CD3^−^ lymphocytes (i.e., non-T cells) CD3^+^ T cells, CD4^+^, TCR-γδ, and CD8^+^ T cells were identified by gating, as shown in Supplementary Figure S1. All of these T-cell subsets and CD3^−^ cells were analyzed for proliferation by dilution of a proliferation dye. Data from one representative animal are shown in Supplementary Figure S2. Figure 2 summarizes the data of experiments with PBMCs from seven different pigs. Live lymphocytes (Figure 2a), CD3^−^ non-T cells (Supplementary Figure S2a), and all major T-cell subsets (Supplementary Figure S2b–e) showed similar reductions on proliferation at DON concentrations higher than 0.4 μM. A significant reduction in proliferation was observed for live lymphocytes (*p* < 0.01), CD3^−^ non-T cells (*p* < 0.05), CD3^+^ T cells (*p* < 0.01), and CD4^+^ T cells (*p* < 0.05) (Figure 2b–e) at 0.8 μM DON. Proliferation of γδ T cells and CD8^+^ T cells was also reduced at 0.8 μM DON (Figure 2f,g), but this was only significant in comparison to no DON, DOM-1, or DON 0.1 µM for γδ T cells (*p* < 0.05) or 0.4 µM DON for CD8^+^ T cells (*p* < 0.05). At 1.6 μM DON, as shown in Figure 2b–g, proliferation was abolished for all cell populations. DOM-1 at 16 μM did not affect proliferation in any cell population (Figure 2b–g).

Next to the proliferation of T-cell subsets, we also aimed to investigate whether DON influences the composition of T-cell subsets that are driven into death by its presence in the context of ConA stimulation. To do so, dead cells were gated (identified by a high fluorescence intensity for the live/dead discrimination dye, Supplementary Figure S3a). In this gate, T-cell subsets (CD4^+^, CD8^+^, and γδ) were identified within total T cells according to the gating shown in Supplementary Figure S1. Due to the high variability in the composition of T-cell subsets between individual pigs, results are displayed as stacked bar charts in Supplementary Figure S3b. T-cell subset composition was compared between T cells stimulated with ConA alone and ConA + 1.6 µM DON, because only at the highest concentration of DON (in the presence of ConA) was the frequency of dead cells significantly increased (Figure 1b). Results showed that for each individual animal, the combination of ConA + 1.6 µM DON resulted in a somewhat higher percentage of γδ T cells (mean increase of 17.6%) whereas CD4^+^ and CD8^+^ T cells were slightly reduced (mean reduction of 11.1 and 5.4%, respectively).

### 2.2. Effect of DON on the Expression of the Activation Marker CD8α and the Costimulatory Molecules CD27 and CD28 within the Major T-cell Subsets

#### 2.2.1. CD8α Expression in the Presence of Different DON Concentrations

**Figure 3:**
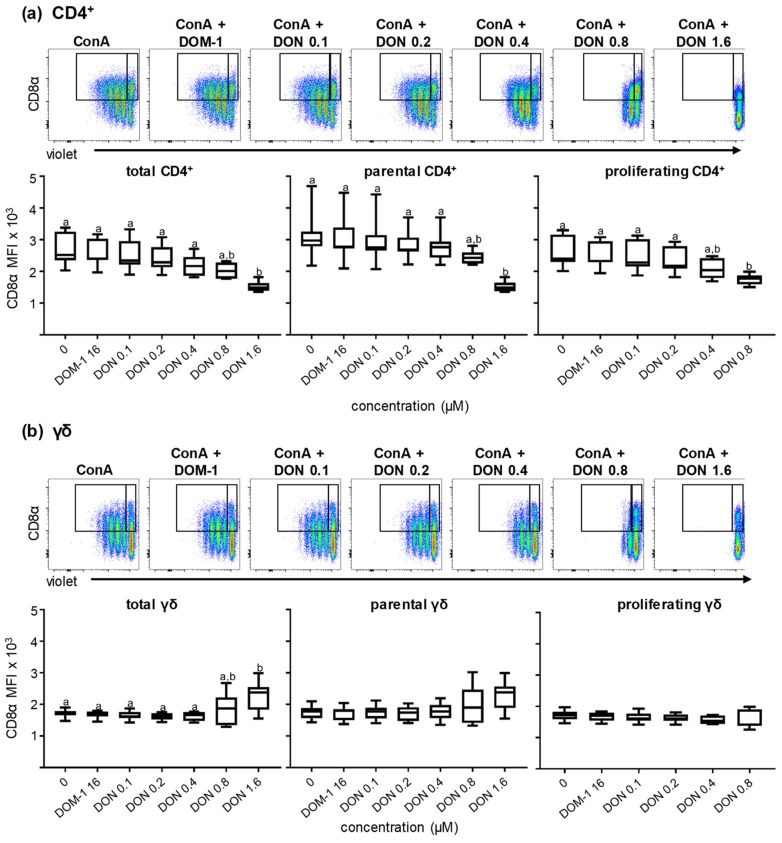
Expression of CD8α in ConA-stimulated and violet proliferation dye-stained CD4^+^ and γδ T cells in the presence of DON and DOM-1. PBMC were cultivated and analyzed by flow cytometry under the conditions described in Figure 2. After gating on lymphocytes and live lymphocytes, CD4^+^ and γδ T-cells were gated within CD3^+^ T cells (Supplementary Figure S1) and analyzed for expression of CD8α and proliferation. Original flow cytometry data shown on the top of (**a**) and (**b**) are from one representative animal. CD8α expression was analyzed by the median fluorescence intensity (MFI) in the total population (both gates together indicated by black rectangles), the parental cell generation (gate on the right), and the proliferating cell generations (gate on the left) for CD4^+^ T cells (a) and γδ T cells (b). The boxplots summarize the results from seven animals and present the expression (MFI) of CD8α within total, parental, and proliferating ConA-stimulated CD4^+^ T cells (a) and γδ T cells (b). Different small letters on boxplots indicate significant differences. (a) Total CD4^+^ T cells a–b: *p* < 0.05, parental CD4^+^ T cells a–b: *p* < 0.01, proliferating CD4^+^ T cells a–b: *p* < 0.05. (b) Total γδ T cells a–b: *p* < 0.05. When small letters are not displayed above the whisker plots, no significant differences were observed (*p* > 0.05).

Next to survival and proliferation, we investigated the effect of DON and DOM-1 on the expression of CD8α within CD4^+^ and γδ T cells in the presence of ConA stimulation. By applying the same methodology as for Figure 2, CD4^+^ and γδ T cells were gated as shown in Figure S1. Subsequently, CD4^+^ T cells and γδ T cells were analyzed for CD8α expression in total, parental, and proliferating cells under the different DON and DOM-1 concentrations (ConA alone, DOM-1 16 μM, DON 0.1, 0.2, 0.4, 0.8, and 1.6 μM). For total and parental (i.e., nonproliferating) CD4^+^ T cells, a significant reduction (*p* < 0.05) in CD8α expression was observed at 1.6 µM of DON (Figure 3a). For proliferating CD4^+^ T cells, a trend for gradual decrease in the expression of CD8α was found at 0.4 µM of DON onwards, although a significant reduction was only reached 0.8 µM of DON. DON 1.6 μM is not indicated in the boxplots showing proliferating populations in Figure 3a,b since at 1.6 μM proliferation was abolished for all T-cell subsets (see Figure 2 and Figure S2b–e). DOM-1 at 16 μM did not affect CD8α expression. Contrary to the CD4^+^ T cells where a reduction of CD8α was observed, in γδ T cells CD8α was affected in the opposite direction (Figure 3b). In total γδ T cells, CD8α expression was significantly increased at 1.6 μM DON (*p* < 0.05). In parental and proliferating γδ T cells this significance level was not reached, even though in the parental generation CD8α expression slightly increased in the highest DON concentration tested (Figure 3b). Again, DOM-1 at the concentration of 16 μM did not affect the median fluorescence intensity (MFI) of CD8α of γδ T cells, for which expression was similar to the levels of the expression when neither DOM-1 nor DON was added into the cultures.

#### 2.2.2. CD28 Expression in the Presence of Different DON Concentrations

**Figure 4:**
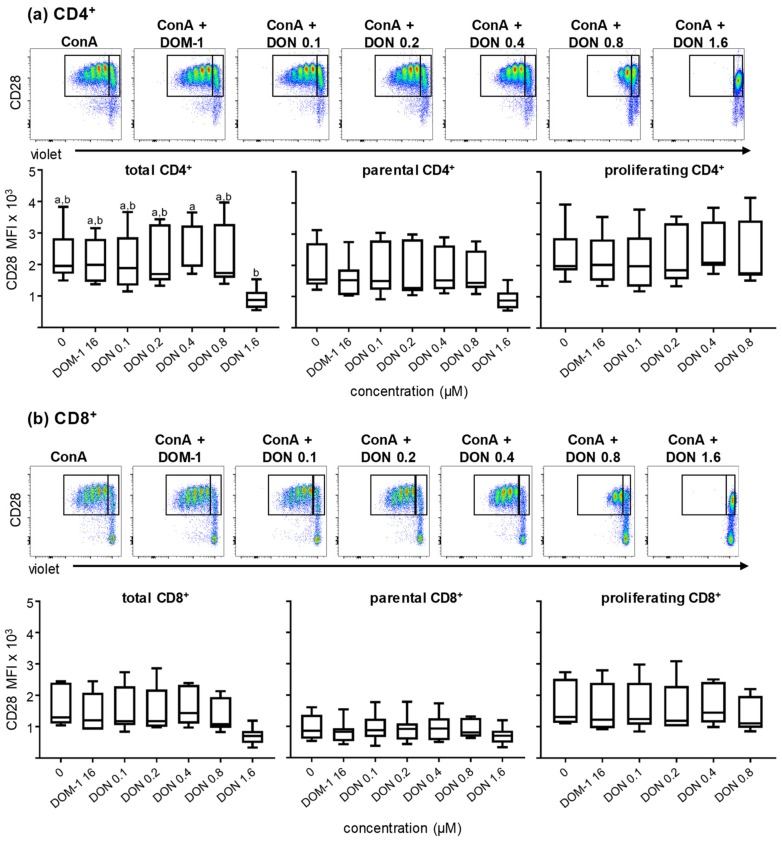
Expression of CD28 in ConA-stimulated and violet proliferation dye-stained CD4^+^ and CD8^+^ T cells in the presence of DON and DOM-1. PBMC were cultivated and analyzed by flow cytometry under the conditions described in Figure 2. After gating on lymphocytes and live lymphocytes, CD4^+^ and CD8^+^ T-cells were gated within CD3^+^ T cells (Supplementary Figure S1) and analyzed for expression of CD28 and proliferation. Original flow cytometry data shown on the top of (**a**) and (**b**) are from one representative animal. CD28 expression was analyzed by the median fluorescence intensity (MFI) in the total population (both gates together indicated by black rectangles), the parental cell generation (gate on the right), and the proliferating cell generations (gate on the left) for CD4^+^ T cells (a) and CD8^+^ T cells (b). The boxplots summarize the results from seven animals and present the expression (MFI) of CD28 within total, parental, and proliferating ConA-stimulated CD4^+^ T cells (a) and CD8^+^ T cells (b). Different small letters on boxplots indicate significant differences. (a) Total CD4^+^ T cells a–b: *p* < 0.05. When small letters are not displayed above the whisker plots, no significant differences were observed (*p* > 0.05).

CD28 is an essential costimulatory molecule for T cells across mammalian species. Recently a monoclonal antibody against porcine CD28 was developed (data currently unpublished) that allowed us to investigate the CD28 expression in CD4^+^ and CD8^+^ T cells under the experimental conditions described above. γδ T cells were excluded from CD28 analysis since in pigs ex vivo only a small subset express this molecule (data currently unpublished). CD4^+^ and CD8^+^ T cells were gated (Figure S1) and analyzed for CD28 expression in total, parental, and proliferating cells under the different DON and DOM-1 concentrations (ConA alone, DOM-1 16 μM, DON 0.1, 0.2, 0.4, 0.8, and 1.6 μM). The MFI of CD28 was downregulated at 1.6 µM DON in total (*p* < 0.05) and parental CD4^+^ T cells, but for the latter this did not reach a significance level of *p* ≤ 0.05 (Figure 4a). CD28 expression in proliferating CD4^+^ T cells was not affected at DON concentrations up to 0.8 µM. Again, the condition of 1.6 µM DON is not shown, since proliferation was completely abolished at this DON concentration. For CD8^+^ T cells (Figure 4b), in all three, total, parental, and proliferating, CD8^+^ T-cell populations, no significant loss of CD28 expression was found, although at at 1.6 μM DON in total CD8^+^ T cells a reduced expression level was observed.

#### 2.2.3. CD27 Expression in the Presence of Different DON Concentrations

**Figure 5:**
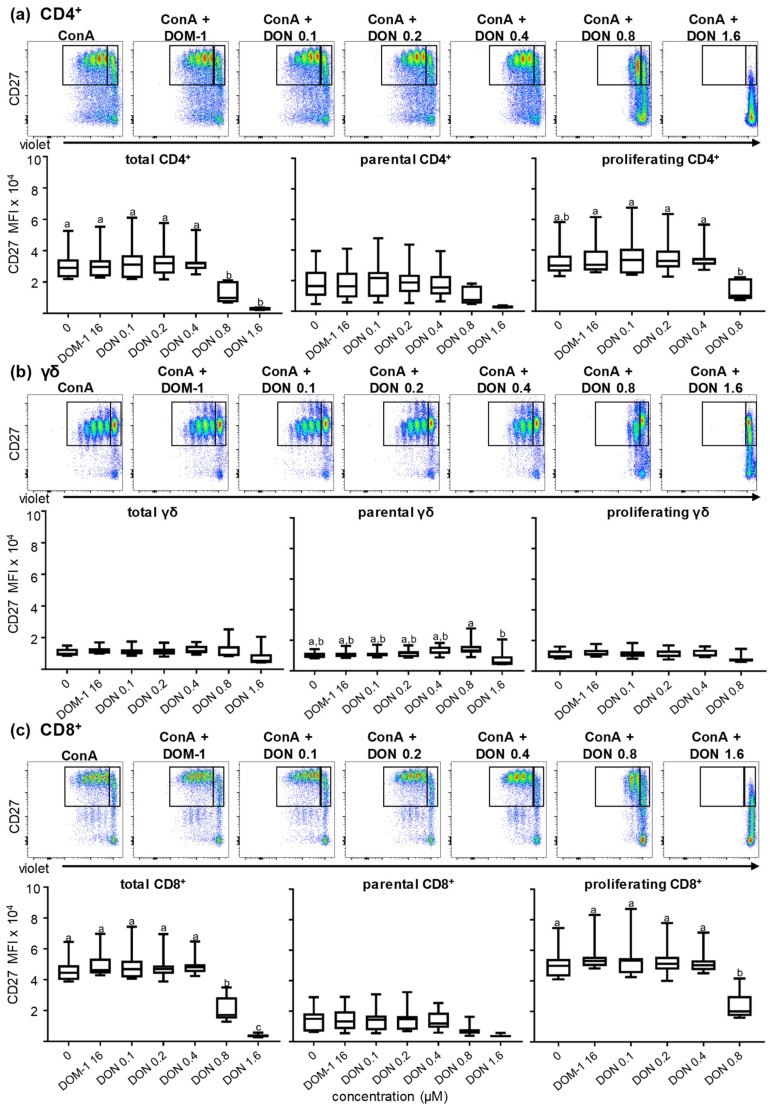
Expression of CD27 in ConA-stimulated and violet proliferation dye-stained CD4^+^, γδ, and CD8^+^ T cells in the presence of DON and DOM-1. PBMC were cultivated and analyzed by flow cytometry under the conditions described in Figure 2. After gating on lymphocytes and live lymphocytes, CD4^+^, γδ, and CD8^+^ T-cells were gated within CD3^+^ T cells (Figure S1) and analyzed for expression of CD27 and proliferation. Original flow cytometry data shown on the top of (a–c) are from one representative animal. CD27 expression was analyzed by the median fluorescence intensity (MFI) in the total population (both gates together indicated by black rectangles), the parental cell generation (gate on the right), and the proliferating cell generations (gate on the left) for CD4^+^ T cells (**a**), γδ T cells (**b**), and CD8^+^ T cells (**c**). The boxplots summarize the results from seven animals and present the expression (MFI) of CD27 within total, parental, and proliferating ConA-stimulated CD4^+^ T cells (**a**), γδ T cells (**b**), and CD8^+^ T cells (**c**). Different small letters on boxplots indicate significant differences. (a) Total CD4^+^ T cells a–b: *p* < 0.05, proliferating CD4^+^ T cells a–b: *p* < 0.05. (b) Parental γδ T cells a–b: *p* < 0.05. (c) Total CD8^+^ T cells a–b: *p* < 0.0001, a–c: *p* < 0.0001, b–c: *p* < 0.05, proliferating CD8^+^ T cells a–b: *p* < 0.01. When small letters are not displayed above the whisker plots, no significant differences were observed (*p* > 0.05).

As outlined in the introduction, CD27 has also been described as an important costimulatory molecule in T cells. In this study, CD27 expression in total, parental, and proliferating CD4^+^, CD8^+^, and γδ T cells was analyzed with the same experimental set-up as described above. As shown by the boxplots of Figure 5a, for CD4^+^ T cells a significant decrease was observed in CD27 expression at DON concentrations of 0.8 μM in total and proliferating cells (*p* < 0.05). This decrease was even higher at 1.6 μM DON for total (*p* < 0.001) CD4^+^ T cells. A decrease in CD27 expression at 1.6 µM of DON was also found for parental CD4^+^ T cells, but this did not reach significance. In γδ T cells (Figure 5b) for all three (total, parental, proliferating) cell populations, CD27 expression was not affected under all different stimulation conditions up to 0.8 µM DON. At 1.6 µM of DON, there was a tendency for a decrease, with a significant difference between parental γδ T cells at 0.8 µM DON and 1.6 µM DON (*p* < 0.05). Data obtained for CD27 expression on CD8^+^ T cells in the presence of different DON concentrations are shown in Figure 5c. In total, parental, and proliferating CD8^+^ T cells, CD27 expression was stable until the concentration of 0.4 μM DON. At 0.8 μM DON a significant decrease was observed in both total (*p* < 0.0001) and proliferating (*p* < 0.01) CD8^+^ T cells, as shown in the boxplots. In total CD8^+^ T cells in 1.6 μM DON the expression of CD27 was further reduced (*p* < 0.05). CD27 expression in the parental CD8 population was less affected, reductions observed at 0.8 and 1.6 μM DON did not reach significance. DOM-1 at a concentration of 16 μM did not affect CD27 expression in any of the three T-cell subsets (Figure 5a–c).

### 2.3. Proliferation of CD27-Defined CD4^+^ and CD8^+^ T-Cell Subsets in the Presence of DON

As mentioned above, CD27 is a costimulatory molecule and in humans, CD27 has been used to characterize the differentiation stage of T cells [36,37,38]. In swine, CD27 expression can be used to identify three CD4^+^ T-cell subsets with distinct functional properties: the CD8α^−^CD27^+^ population represents naïve CD4^+^ T cells, the CD8α^+^CD27^+^ population has functional properties of central memory T helper cells, and the CD8α^+^CD27^−^ population has functional traits of effector memory T cells [26]. In porcine CD8^+^ T cells, the differentiation stages following antigen encounter have not been investigated in so much detail. Talker et al. proposed that perforin in combination with CD27 could describe different developmental stages of porcine CD8^+^ T cells. Perforin^−^CD27^+^ CD8^+^ T cells were proposed to represent naïve CD8^+^ T cells, whereas perforin^+^CD8^+^ T cells might be distinguished in early (CD27^dim^ phenotype) and late effector cells (CD27^−^) by the expression of CD27 [27].

**Figure 6:**
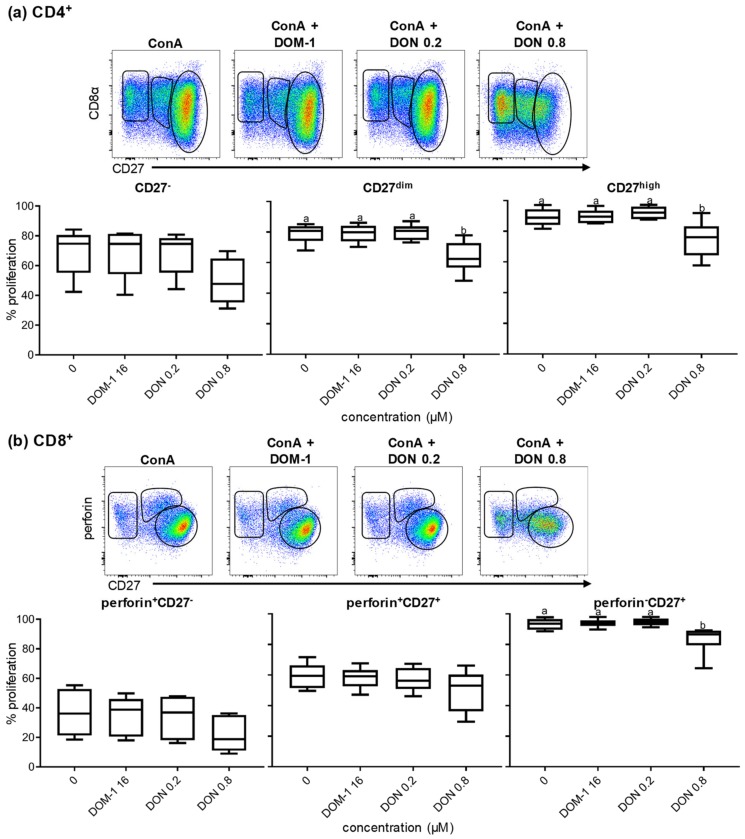
Proliferation of CD27-defined CD4^+^ T-cell subpopulations and perforin/CD27-defined CD8β^+^ T-cell subpopulations in the presence of DON and DOM-1. PBMCs were stained with violet proliferation dye. After four days of in vitro cultivation in the presence of ConA alone or in combination with DON (0.2, 0.8 μM) and DOM-1 (16 μM) they were harvested and subjected to flow cytometry. Lymphocytes were gated by light scatter properties and further subgated for exclusion of dead cells with a live/dead discrimination dye (not shown). (**a**) CD4^+^ cells were gated within live lymphocytes and then further subgated for CD27^−^ (cells gated on the left), CD27^dim^ (cells gated in the middle), and CD27^+^ cells (cells gated on the right), as shown on the first row (data from one representative animal). The boxplots show the percentage of proliferating cells of six different animals under different stimulation conditions. Different small letters on boxplots indicate significant differences among the conditions (CD27^dim^: *p* < 0.01, CD27^high^: *p* < 0.05). (**b**) CD8β^+^ cells were gated within live lymphocytes and then further subgated for CD27^−^perforin^+^ (cells gated on the left), CD27^+^perforin^+^ (cells gated on the middle), and CD27^+^perforin^−^ cells (cells gated on the right), as shown on the first row (data from one representative animal). The boxplots show the percentage of proliferating cells of six different animals. Different small letters on boxplots indicate significant differences among the conditions (CD27^+^perforin^−^: *p* < 0.05). (a,b) When small letters are not displayed above the whisker plots, no significant differences were observed (*p* > 0.05).

In the present study, we investigated the influence of two DON concentrations (0.2 and 0.8 μM) and DOM-1 (16 µM) on CD27-defined CD4^+^ and CD8^+^ T-cell subsets introduced above. The DON concentration of 0.8 µM was chosen because at this dose the proliferation of CD4^+^ and CD8^+^ T cells was already impaired but not completely abolished, as with 1.6 µM of DON (Figure 2). DON at a concentration of 0.2 µM and DOM-1 (16 µM) were considered as putative negative controls. After a violet proliferation dye staining and four days of cultivation, cells were harvested and analyzed. In one set of samples CD4^+^ cells were gated (not shown) and then further subgated based on three different levels of CD27 expression: CD27^−^, CD27^dim^, and CD27^high^ (Figure 6a, pseudocolor plots on top). Different from the phenotypic description of CD4^+^ T cells given above, which also includes CD8α, we excluded this molecule from the gating strategy since an upregulation of CD8α occurs on porcine CD4^+^ T cells upon ConA stimulation [25]. The boxplots in Figure 6a summarize the data of CD4^+^ T cells from six animals and show the proliferation of each CD27-defined subset under the stimulation of ConA and different DON concentrations. Representative violet proliferation data from a single animal are shown in Supplementary Figure S4a–c). In the ConA-only stimulation condition (no DON or DOM-1), the highest frequency in proliferation among all three subsets was observed in the CD27^high^ population (mean frequency of proliferation was 90%) and the lowest for the CD27^−^ population (frequency of proliferation < 80%). Proliferation was not affected at 16 μM of DOM-1 and 0.2 μM of DON, but was significantly decreased at 0.8 μM DON for both CD27^high^ and CD27^dim^ populations (*p* < 0.05 and *p* < 0.01, respectively). The CD27^−^ population (Figure 6a) showed a decrease in proliferation at DON 0.8 μM as well, but this difference did not reach significance.

Similarly, CD8^+^ T cells were sub-gated into three populations depending on their perforin and CD27 expression (perforin^+^CD27^−^, gated on the left; perforin^+^CD27^+^, gated in the middle; perforin-CD27^+^, gated on the right) and analyzed for their proliferation capacities (Figure 6b). Proliferation of each subset is shown in the boxplots, summarizing results of CD8^+^ T cells from six animals. Representative violet proliferation data from a single animal are shown in Supplementary Figure S4d–f). The lowest capacity of proliferation was observed for the perforin^+^CD27^−^ population and the perforin^+^CD27^+^ CD8^+^ T-cell population, where the mean frequencies of proliferation under ConA-only stimulation were approximately 40% and 60%, respectively. The perforin^−^CD27^+^ population, presumably representing naïve CD8^+^ T cells, showed the highest proliferation rate under ConA-only stimulation, with frequencies that reached almost 100%. Proliferation of all three subsets was decreased in the presence of DON 0.8 μM, but reached significance only for the perforin^−^CD27^+^ CD8^+^ T-cell subset (*p* < 0.05). DOM-1 (16 μM) and DON 0.2 μM did not affect proliferation in all three perforin/CD27-defined CD8^+^ T-cell subsets.

### 2.4. Phenotypic Changes in CD8α and CD27 Expression in Proliferating Sorted and Unsorted Naïve CD4^+^ T Cells in the Presence of DON

**Figure 7:**
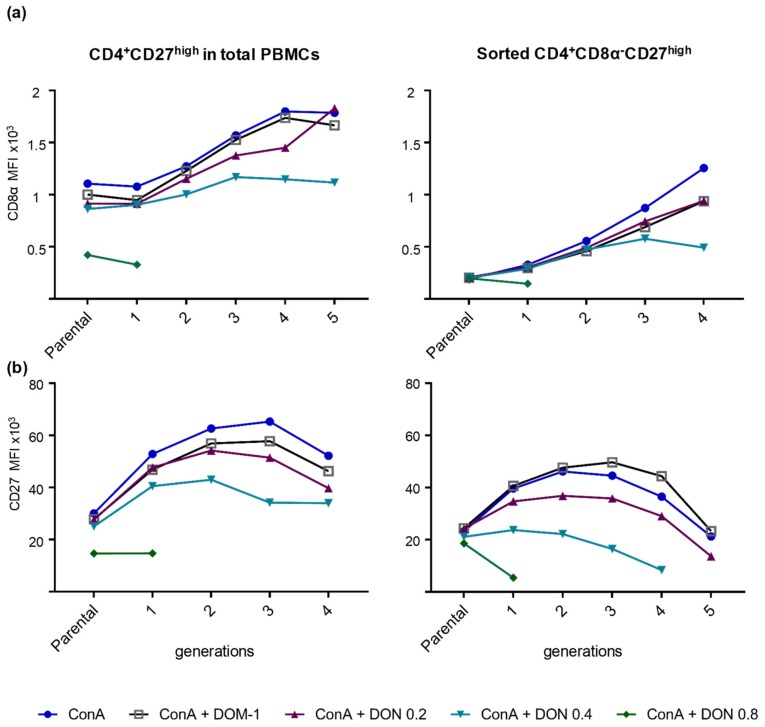
Expression of CD8α and CD27 on sorted CD8α^−^CD27^high^ (naïve) CD4^+^ T cells in the presence of DON and DOM-1. Total PBMCs and sorted CD8α^−^CD27^high^ CD4^+^ T cells were stained with violet proliferation dye and stimulated with either: ConA alone (blue circles), ConA + DOM-1 (open grey squares), ConA + 0.2 µM DON (purple triangles), ConA + 0.4 µM DON (turquoise triangles), or ConA + 0.8 µM DON (green diamonds). After four days of in vitro cultivation, cells were stained for CD4, CD8α, and CD27, and analyzed for CD8α and CD27 expression by the median fluorescence intensity (MFI) in each proliferating generation of CD4^+^ T cells following a gating, illustrated in Supplementary Figure S4b. The graphs show the expression of CD8α in bulk culture-derived (left) and sorted CD4^+^CD27^high^ (right) cells (**a**) and the expression of CD27 in bulk culture-derived (left) and sorted CD4^+^CD27^high^ (right) cells (**b**). The x-axis indicates the parental generation (nonproliferating cells) and the number of divisions (generations) that the proliferating cells have undergone. The data shown are from one representative animal out of three.

The results displayed in Figure 6 and Supplementary Figure S4 indicate that CD27^high^ CD4^+^ and CD8^+^ T cells were most affected in their proliferation by a DON dose of 0.8 µM. Based on previous observations [26,27], these phenotypes most probably represent naïve T cells. Hence, this finding suggested that naïve T cells are the most severely affected T-cell subset by DON. However, since these results were obtained in bulk cultures of PBMCs, it is conceivable that cytokines produced by other cell types (e.g., more differentiated T cells that respond with effector cytokine production to ConA stimulation [26]) influenced these results. To investigate this further, we sorted naïve CD4^+^ T cells based on a CD8α^−^CD27^high^ phenotype (sort purity shown in Supplementary Figure S5a). After sorting, sorted and unsorted (total PBMCs) cells were subjected to a violet proliferation staining and were cultivated in the presence of ConA, without or with 0.2, 0.4, and 0.8 μM DON, as well as 16 μM DOM-1. Sorted CD8α^−^CD27^high^ CD4^+^ T cells were also cocultivated with CD172a^+^ monocytes in a 10:1 ratio, which in previous studies was found to support their proliferation [26]. After four days, the cells were harvested and restained for the same markers as used for the initial sorting and gated as depicted on Supplementary Figure S5b. Subsequently, the MFIs of CD8α and CD27 were analyzed in the parental population and in each proliferating generation of CD4^+^CD27^high^ unsorted and sorted cells for all different DON/ DOM-1 concentrations (Supplementary Figure S6 and Figure 7). For both sorted and unsorted naïve CD4^+^ T cells, CD8α expression was upregulated in all generations of proliferating cells stimulated with ConA alone or in combination with DOM-1 and DON 0.2 μM. In the presence of ConA + 0.4 μM DON, CD8α expression in both sorted and not sorted cells was initially slightly upregulated until the third generation, but was reduced afterwards (Figure 7a). For CD4^+^ T cells stimulated with ConA + 0.8 μM DON, a slight downregulation was observed in CD8α expression between the parental and the sole descendant generation. Regarding CD27 expression (Figure 7b), an upregulation was initially observed in the first generation for both sorted naïve (CD8α^−^CD27^high^) CD4^+^ T cells and not sorted CD27^high^ CD4^+^ T cells stimulated with DOM-1 and 0.2 and 0.4 μM DON. However, for the sorted naïve CD4^+^ T cells, a downregulation was already observed after the second proliferating generation. This applied to all DON and DOM-1-stimulated cells, with a stronger reduction being observed in cells stimulated with ConA + 0.4 μM DON. For naïve sorted cells stimulated with 0.8 μM DON a downregulation of CD27 expression was observed at the first and only proliferating generation (Figure 7b). Overall, the results for CD8α and CD27 expression levels matched those ones described in Figure 3a and Figure 5a, but both molecules had lower expression levels in sorted naïve CD4^+^ T cells than CD4^+^ T cells in the bulk culture.

## 3. Discussion

Numerous studies that have been conducted in recent years have demonstrated the negative effect of DON on human, murine, bovine, and porcine cells in vitro [2,9,11,29,32,33,35]. In vitro studies on human, murine, and porcine cells have shown that DON reduces the cell viability [29,32,33,39,40]. In our study, we investigated the influence of DON on porcine lymphocytes. Similar to other studies where lymphocytes and other cell types were studied, an increase of dead cells was observed in medium and ConA-stimulated lymphocytes at DON concentrations higher than 0.8 μM (Figure 1) [11,29]. The impact of DON on cell proliferation has been studied as well in the past [11,12,13,35]. Novak et al., Taranu et al., and Daenicke et al. demonstrated a loss in lymphocyte proliferation at DON concentrations higher than 0.4 μM [11,12,13]. None of the studies investigating the impact of DON on cell proliferation in pigs have focused though on the impact of DON on proliferation of the major T-cell subsets, namely CD4^+^, CD8^+^, and γδ T cells. In the present study, we analyzed the proliferation of these T-cell subsets in the presence of various DON concentrations. All major T-cell subsets were similarly affected and a loss in proliferation could be observed at DON concentrations higher than 0.4 μM, which is in accordance with Novak et al., where bovine T-cell subsets were studied [13] (Figure 2).

The costimulatory molecules CD27 and CD28 are pivotal for optimal T-cell activation and for the survival and proliferation of activated T cells [16,20,21,41,42]. CD27 promotes the survival of activated T cells and contributes to the maintenance of memory T cells. In addition, it has been described that CD27 plays an important role in the accumulation of antigen-specific CD8^+^ T cells at the site of infection [14,15,18]. CD28 as well, regulates cell survival by the induction of the Bcl-X_L_ protein and enhances the proliferation of T cells either via an IL-2-dependent or independent manner [22,23,43]. For porcine CD4^+^ T cells, CD8α has been described as an activation marker, which is upregulated upon antigen encounter in vivo, and hence can be used for the identification of antigen-experienced cells [25,27,44]. Since CD8α is upregulated after stimulation, we hypothesized that its expression could be affected by exposure to DON during porcine T-cell proliferation. The analysis revealed that CD8α upregulation in CD4^+^ T cells was reduced at DON concentrations higher than 0.4 μM, but was increased for the highest DON concentration tested (1.6 µM) in total and parental γδ T cells (Figure 3). CD27 expression was significantly downregulated in both CD4^+^ and CD8^+^ T cells at DON concentrations higher than 0.4 μM, but not in γδ T cells (Figure 5). On the contrary, CD28 was less affected by DON and showed only a downregulation at 1.6 μM DON in total CD4^+^ and CD8^+^ T cells (Figure 4). This negative impact of DON on CD8α and CD27 expression levels could lead to a partial impairment of T-cell stimulation and a suboptimal T-cell response following vaccination or infection of pigs. Nevertheless, since there is a certain degree of redundancy in the role of CD27 and CD28 in the costimulation of T cells, the finding that DON does impair CD28 expression only at the highest tested DON concentration suggests that a certain degree of T-cell responsiveness still remains, probably also under in vivo conditions.

We also analyzed whether DON influences the composition of T-cell subsets that are driven into death by its presence in the context of ConA stimulation. The overall composition of the T-cell subsets was very variable between animals, reflecting the high variability that is seen in general in the numbers of different T-cell subsets in outbred pigs of about six months of age [27]. The combination of ConA + 1.6 µM DON resulted in higher percentages of γδ T cells, whereas CD4^+^ and CD8^+^ T cells were slightly reduced. This suggests that CD4^+^ and CD8^+^ T cells were more prone for cell death in the presence of ConA and DON than γδ T cells. This conclusion is based on the observation that at 1.6 µM DON T-cell proliferation was completely abolished (Figure 3d–g), hence, it can be excluded that γδ T cells expanded by proliferation prior to death. Of note, this coincides with the observation that for the DON-induced reduction in CD8α and CD27 expression levels of γδ T cells were also less affected than CD4^+^ and CD8^+^ T cells (Figure 3, Figure 5, respectively, see also above).

CD27 has also been described as a molecule in humans and swine that can be used to define and separate naïve T cells from central memory and effector memory T cells. Depending on the expression levels of CD27, discrete CD4^+^ and CD8^+^ T-cell subsets have been characterized [26,27,36,37,38,45]. As outlined above, for both T-cell subsets, we observed a reduction in CD27 expression levels from 0.8 µM of DON onwards (Figure 5a,c), but despite this reduction it was still possible to separate CD4^+^ T cells into CD27^high^, CD27^dim^, and CD27^−^ cells (Figure 6a, top). The same applied to CD8^+^ T cells where we applied a gating strategy established for ex vivo CD8^+^ T cells to differentiate naïve (CD27^high^perforin^−^), early effector (CD27^dim^perforin^+^), and putative late effector/memory (CD27^−^perforin^+^) CD8^+^ T cells ([27,46]; Figure 6b top). Our assumption is that for both T-cell subsets, CD27^high^ cells after four days of in vitro cultivation in the presence of ConA (with or without DON) still represent T cells that were in a naïve state at the start of cultivation. Indeed, a relatively stable expression of CD27 in sorted CD4^+^CD8α^+^CD27^high^ cells after four days of ConA stimulation was shown in previous work from our laboratory [26]. In accordance with these previously published data for porcine CD4^+^ T cells, T cells with CD27^dim^ and CD27^−^ phenotypes proliferated less than the naïve T cells (CD27^high^) when they were stimulated only with ConA. During the additional presence of 0.8 µM of DON, our data revealed that the reduction of CD27 expression (Figure 5a) coincides with significantly decreased proliferation in particular in CD27^high^ CD4^+^ T cells (Figure 6a). Analogous results were obtained for CD8^+^ T-cell subsets (Figure 6b.), i.e., the proliferation of putatively naïve CD8^+^ T cells was most affected at 0.8 μM DON, with a significant decrease being observed. Hence, these data suggest that in both CD4^+^ and CD8^+^ T-cell subsets, which at the start of the cultivation period had a naïve phenotype, proliferation was most impaired, which might be partially caused by the reduced expression of the costimulatory molecule CD27. Under in vivo conditions, these cells would be involved in the initiation of primary immune responses against pathogens encountered for the first time.

The prominent effect of DON on naïve T-cell proliferation motivated us to investigate whether such an effect would be even more prominent under the applied in vitro conditions in sorted naïve T cells. The outcome regarding CD8α and CD27 expression in sorted naïve CD4^+^ T cells was similar to the results that we obtained from CD4^+^ T cells in bulk culture (Figure 7). CD8α expression was upregulated in all proliferating generations of cells stimulated with ConA alone or in combination with DOM-1 and 0.2 μM DON. Sorted naïve CD4^+^ T cells stimulated with ConA + 0.4 μM DON or 0.8 μM DON showed a slight CD8α downregulation after the third generation and first generation, respectively. Regarding CD27 expression in sorted naïve CD4^+^ T cells, a slight upregulation was observed in the first generations, but was followed by a downregulation for later generations of stimulated CD4^+^ T cells, regardless of the presence of DON. These data are in accordance with data published from Reutner et al., where CD8α expression was strongly upregulated in sorted naïve CD4^+^ T cells, whereas CD27 showed a minor downregulation [26]. Nevertheless, both in the bulk culture and in sorted CD4^+^ T cells, DON reduced the overall CD27 expression levels across the majority of different cell generations. Overall, the results withdrawn from the sorting experiments are very similar to the results obtained from the CD4^+^ T cells in the bulk culture. This suggests that naïve T cells, which have the highest capacity for proliferation, are indeed most susceptible to the effects of DON, affecting both their proliferation and expression of the costimulatory molecule CD27.

Finally, in all the assays performed in this study, the DON-derivative DOM-1 did not show any of the impairments on proliferation or molecule expression levels that were observed with DON concentrations ≥0.4 µM, despite the fact that DOM-1 was used in a 10-fold higher concentration than the highest DOM concentration (16 µM and 1.6 µM, respectively). This further corroborates the current notion that the structural changes in DOM-1 indeed lead to a substantial reduction of the biological effects triggered by DON [29,30,31]. It should be noted that a concentration of 357 µM DOM-1 has been reported to reduce ConA-induced proliferation of porcine and chicken PBMC [13]. However, the relevance of this concentration is debatable, as on one hand, 357 µM DOM-1, which corresponds to ~100 ppm DON in feed, is very high and is not found in finished pig feed, as recently shown [47]. On the other hand, 0.1–0.2% impurities of DON (corresponding to 0.357–0.714 µM) were present in the DOM-1 stock used by Novak et al., 2018 [13]. This was reported in Mayer et al., 2017 [31]). Putting this into the context of our findings, where DON concentrations of 0.8 µM resulted already in significant reductions in the proliferation of T-cell subsets, it is likely that the reduction described by Novak et al. 2018 [13] was caused by this impurity.

In summary, our study provides, for the first time, information on how DON negatively affects the proliferation of the major porcine T-cell subsets and the expression of the costimulatory molecules CD27 and CD28, which are required for optimal T-cell activation, survival, and proliferation. The observed downregulation of CD27 and the loss in proliferation that DON causes under the conditions applied here may also apply in vivo. This could lead to compromised immune responses, in particular to pathogens that require an activation of the adaptive immune system for their control. If such microorganisms are encountered for the first time, an activation of naïve T cells is required, which according to our results seem to be most susceptible to DON.

## 4. Materials and Methods

### 4.1. Animals and Cell Isolation

Heparinized blood was collected from 6-month-old healthy pigs from an abattoir after anesthetizing them with high electric voltage and followed by exsanguination, which is in accordance to the Austrian Animal Welfare Slaughter Regulation.

PBMCs, used for the methods described below, were isolated after centrifugation for 30 min at 920× *g* on Ficoll-Paque gradient (LSM/Pancoll human, density: 1.077 g/mL, PAN Biotech, Aidenbach, Germany), as described before [48]. Cells were counted by using a cell counter (XP-300 Haematology Analyser, Sysmex Austria GmbH, Vienna, Austria) before cryopreserving them at −150 °C (as described in Leitner et al. [49]) for future use.

### 4.2. Proliferation Assays

PBMCs were thawed and counted in phosphate-buffered saline (PBS, PAN Biotech GmbH, Aidenbach, Germany) and then labelled with the CellTrace^TM^ Violet Cell Proliferation Kit (Thermo Fisher Scientific, Waltham, MA, USA), as described elsewhere [13,19]. Briefly, the cell number was adjusted to 2 × 10^7^ in 1 mL and was mixed with 1 mL of violet dye solution (stock solution concentration 5 μM) and incubated for 10 min in a water bath at 37 °C. In order to stop the uptake of the violet dye, 2 mL of fetal calf serum (FCS) (PAN-Biotech GmbH) were added and incubated for 15 min in the dark. Finally, after washing the cells in culture medium three times (RPMI 1640 + 10% heat-inactivated FCS + 100 U/mL penicillin, 100 µg/mL streptomycin, all from PAN-Biotech), the cells were counted and 5 x 10^5^ cells per well were plated in 96-well round-bottom plates (Greiner Bio-One, Frickenhausen, Germany). Cells were either cultivated in medium or stimulated and cultivated with ConA (3 µg/mL, Amersham Biosciences, Uppsala, Sweden) in the presence or absence of different deoxynivalenol (DON) concentrations (0.1, 0.2, 0.4, 0.8, and 1.6 µM) and a single deepoxy-deoxynivalenol (DOM-1) concentration (16 µM) at 37 °C for four days. In each well the final volume was 200 μL. DON and DOM-1 were initially dissolved in sterile water to obtain a stock solution of 5 mM and stored at −20 °C. Both substances were provided from Biopure, Romer Labs^®^, Tulln, Austria and had a purity of ≥99%.

### 4.3. Flow Cytometry Staining and Antibodies

After four days of in vitro cultivation at 37 °C, cells were harvested and washed in PBS + 3% FCS. The antibodies (Abs) listed in Table 1 were used for cell surface staining, and afterwards cells were analyzed for both proliferation and surface marker expression in a six-color staining by flow cytometry. Cells were first stained with primary and then with secondary Abs and incubated for 20 min at 4 °C in 96-well round bottom plates. During the incubation with secondary Abs, the viability dye VDeFluor780 (Thermo Fisher) was included. Between the two incubations, the cells were washed twice with PBS. After the last incubation, the cells were washed in PBS + 3% FCS and were analyzed in 200 μL of the same buffer by a FACSCanto II flow cytometer (BD Biosciences, San Jose, CA, USA). At least 2 × 10^5^ lymphocytes were recorded per sample. The obtained flow cytometry data were further analyzed by FlowJo software version 10.5.3 (FlowJo LLC, Ashland, OR, USA).

For an additional set of experiments, an intracellular staining of perforin was performed, by three additional incubation steps. Following the incubation with the secondary reagents and after washing two times with PBS only, the free binding sites of the secondary Abs were blocked by incubation with mouse IgG molecules (2 μg per sample, ChromPure, Jackson ImmunoResearch, West Grove, PA, USA). During the incubation with the IgG molecules, the viability dye VDeFluor780 (Thermo Fisher) was included. Following an incubation of 20 min and 2 washing steps with PBS + 3% FCS, the cells were fixed and permeabilized with eBioscience^TM^ Foxp3/Transcription factor staining buffer set (Thermo Fisher). After this incubation, cells were incubated with perforin-specific Abs for 30 min at 4 °C [27,28].

### 4.4. Fluorescence-Activated Cell Sorting (FACS) and Antibodies

For sorting of naïve (CD8α^−^CD27^high^) CD4^+^ T cells and CD4^−^CD172a^+^ antigen-presenting cells (APCs) on a FACSAria (BD Biosciences), approximately 7 × 10^8^ PBMCs were thawed and resuspended in 40 mL sorting medium, consisting of RPMI 1640 (PAN-Biotech) supplemented with 5% heat-inactivated FCS (Sigma-Aldrich, St Louis, MO, USA), 5% heat-inactivated porcine plasma (in-house prepared), and 2mM EDTA [28]. The cells were stained with the primary and secondary Abs indicated in Table 2. The purity of the CD4^+^CD27^high^ population was ≥ 97%, as shown in Supplementary Figure S5.

### 4.5. Proliferation Assay and In Vitro Cultivation of Sorted T Cells

Sorted naïve T cells and total PBMCs were labelled using the CellTrace^TM^ Violet Cell Proliferation Kit (Thermo Fisher Scientific, Waltham, MA, USA), as described above and in [26,50]. After the violet proliferation labelling, sorted CD8α^−^CD27^high^ CD4^+^ T cells and total PBMCs were counted and 2 × 10^5^ cells per well were plated in triplicates in 96-well plates (Greiner Bio-One, Kremsmünster, Austria). The cells were resuspended and seeded either in cell culture medium alone (RPMI 1640 supplemented with 10% heat-inactivated FCS, 100 IU/mL penicillin, and 100 µg/mL streptomycin) or stimulated with one of the following stimulation conditions: (i) ConA alone (3 µg/mL, Amersham Biosciences), (ii) ConA + DON 0.2 μM, (iii) ConA + DON 0.4 μM, (iv) ConA + DON 0.8 μM, and (v) ConA + DOM-1 16 μM. In addition, sorted CD172a^+^ APCs were added to all wells with sorted CD8α^−^CD27^high^ CD4^+^ T cells in a 1:10 ratio (2 × 10^4^ cells/well), as well as recombinant porcine IL-2 (50 ng/mL, R&D Systems, Minneapolis, MN, USA). Final total volume per well was 200 μL. After four days of in vitro cultivation cells were harvested, washed in PBS + 3% FCS, stained with the same Abs as prior to sorting (CD4, CD8α, and CD27), and analyzed on a FACSAria (BD Biosciences). At least 1 × 10^5^ cells were recorded per sample.

### 4.6. Statistical Analysis

Descriptive statistics were performed by using GraphPad Prism V7.04 (GraphPad Software, San Diego, CA, USA). The data sets were subjected to multiple comparison tests with one-way ANOVA and Bonferroni’s multiple comparisons test. The following levels of significance were defined: *p* ≤ 0.05, *p* ≤ 0.01, *p* ≤ 0.001, and *p* ≤ 0.0001.

## Figures and Tables

**Table 1:** Labeling reagents for general immunophenotyping.

Antigen	Clone	Isotype	Fluorochrome	Labeling Strategy	Source of Primary Ab
**Analysis of co-Stimulatory Molecules**					
CD3	BB23-8E6-8C8	IgG2a	PerCP-Cy5.5	Directly conjugated	BD Biosciences
CD4	74-12-4	IgG2b	FITC	Directly conjugated	BD Biosciences
CD8α	11/295/33	IgG2a	Alexa647	Directly conjugated	In house
TCR-γδ	PPT16	IgG2b	BV605	Biotin-Streptavidin ^3^	In house
CD28 ^1^	3D11 ^2^	IgG1	PE	Secondary antibody ^4^	In house ^2^
CD27 ^1^	b30c7	IgG1	PE	Secondary antibody ^4^	In house
**Analysis of Perforin, CD27 and CD8α Expression**					
CD27	b30c7	IgG1	Alexa647	Secondary antibody ^5^	In house
CD4	74-12-4	IgG2b	FITC	Secondary antibody ^6^	In house
CD8α ^1^	11/295/33	IgG2α	PE-Cy7	Secondary antibody ^7^	In house
CD8β ^1^	PG164a	IgG2a	PE-Cy7	Secondary antibody ^7^	In house
Perforin	δ-G9	IgG2b	PerCP-eFluor710	Directly conjugated	eBioscience

^1^ Stained in parallel samples. ^2^ Kindly provided by Niklas Beyersdorf, University of Würzburg, Germany. ^3^ Streptavidin-BV605, BioLegend, San Diego, CA, USA. ^4^ Goat anti-mouse IgG1-PE, Southern Biotech, Birmingham, AL, USA. ^5^ Goat anti-mouse IgG1-Alexa647, Thermo Fisher. ^6^ Goat anti-mouse IgG2b-FITC, ThermoF isher. ^7^ Goat anti-mouse IgG2a-PE-Cy7, Southern Biotech.

**Table 2:** Labeling reagents for FACS.

Antigen	Clone	Isotype	Fluorochrome	Labeling Strategy	Source of Primary Ab
**Staining for Cell Sorting**					
CD4	74-12-4	IgG2b	FITC	Directly conjugated	BD Biosciences
CD27	b30c7	IgG1	PE	Secondary antibody ^1^	In house
CD8α	11/295/33	IgG2a	Alexa647	Secondary antibody ^2^	In house
CD172a	77-22-15A	IgG2b	eFluor450	Biotin-Streptavidin ^3^	In house

^1^ Goat anti-mouse IgG1-PE, Southern Biotech. ^2^ Goat anti-mouse IgG2a-Alexa647, Thermo Fisher. ^3^ Streptavidin-eFluor450, Thermo Fisher.

## Supplementary Materials

The following are available online at https://www.mdpi.com/2072-6651/11/11/644/s1, Figure S1: Gating strategy of lymphocytes and major T-cell subsets derived from blood, Figure S2: Proliferation of CD3^−^ cells and major T-cell subsets in the presence of DON and DOM-1, Figure S3. Relative distribution of CD4^+^, CD8^+^, and γδ T cells within dead CD3^+^ T cells. Figure S4: Proliferation of CD27-defined CD4^+^ and perforin/CD27-defined CD8^+^ T-cell subsets in the presence of DON and DOM-1, Figure S5: Gating strategy before and after sorting of naïve CD4^+^ T cells, Figure S6: Influence of DON and DOM-1 on the expression of CD8α and CD27 in ConA-stimulated sorted naïve CD4^+^ T cells and CD4^+^ T cells present in a PBMC bulk culture.
