# Supplementary material for: Deoxynivalenol Affects Proliferation and Expression of Activation-Related Molecules in Major Porcine T-Cell Subsets
Source: Toxins (Basel). 2019 Nov 5;11(11):644. doi: 10.3390/toxins11110644 (PMC6891462; doi:10.3390/toxins11110644)
Supplement: Supplementary file 1 [file toxins-11-00644-s001.pdf]

Supplementary Materials: Deoxynivalenol Affects Proliferation and Expression of Activation-Related Molecules in Major Porcine T-cell Subsets

Eleni Vatzia, Alix Pierron, Armin Saalmüller, Elisabeth Mayer and Wilhelm Gerner *

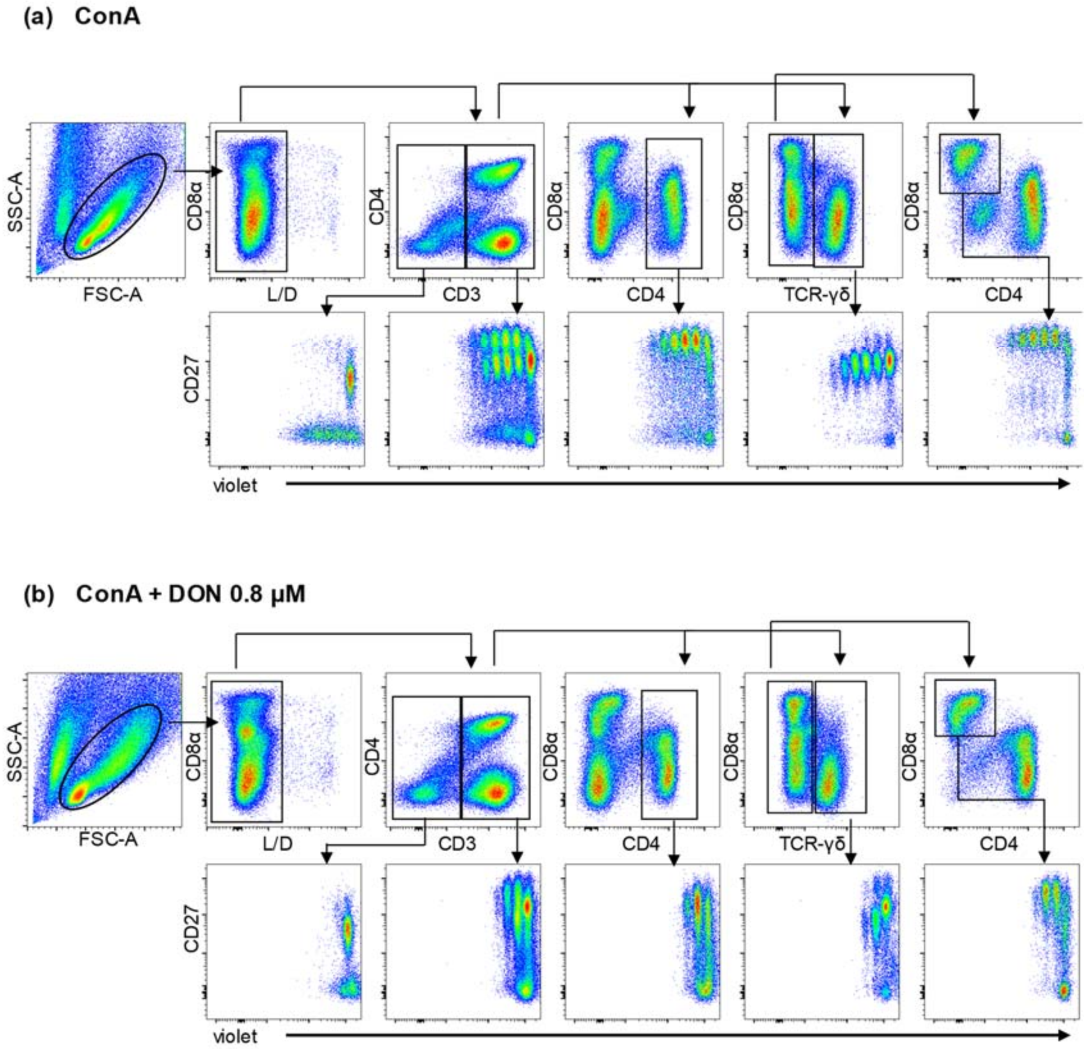

Figure S1. Gating strategy of lymphocytes and major T-cell subsets derived from blood. Representative data after in vitro cultivation of PBMCs for four days in the presence of ConA (**a**) or ConA + DON 0.8 µM (**b**) are shown. Lymphocytes were gated according to their light scatter properties, followed by gating on live lymphocytes to exclude dead cells. Live lymphocytes were separated into $CD3^-$ non T-cells and $CD3^+$ T cells. Following $CD3^+$ gating, cells were sub-gated for $CD4^+$, TCR-$\gamma\delta^+$ and TCR-$\gamma\delta^-$ cells. $CD8\alpha^{high}$ T cells were gated within $CD3^+$TCR-$\gamma\delta^-$ cells. The second row shows CD27 expression in combination with proliferation of all subsets that were gated ($CD3^-$ non-T cells, $CD3^+$, $CD4^+$, TCR-$\gamma\delta^+$, TCR-$\gamma\delta^-$ and $CD8\alpha^{high}$ T cells).

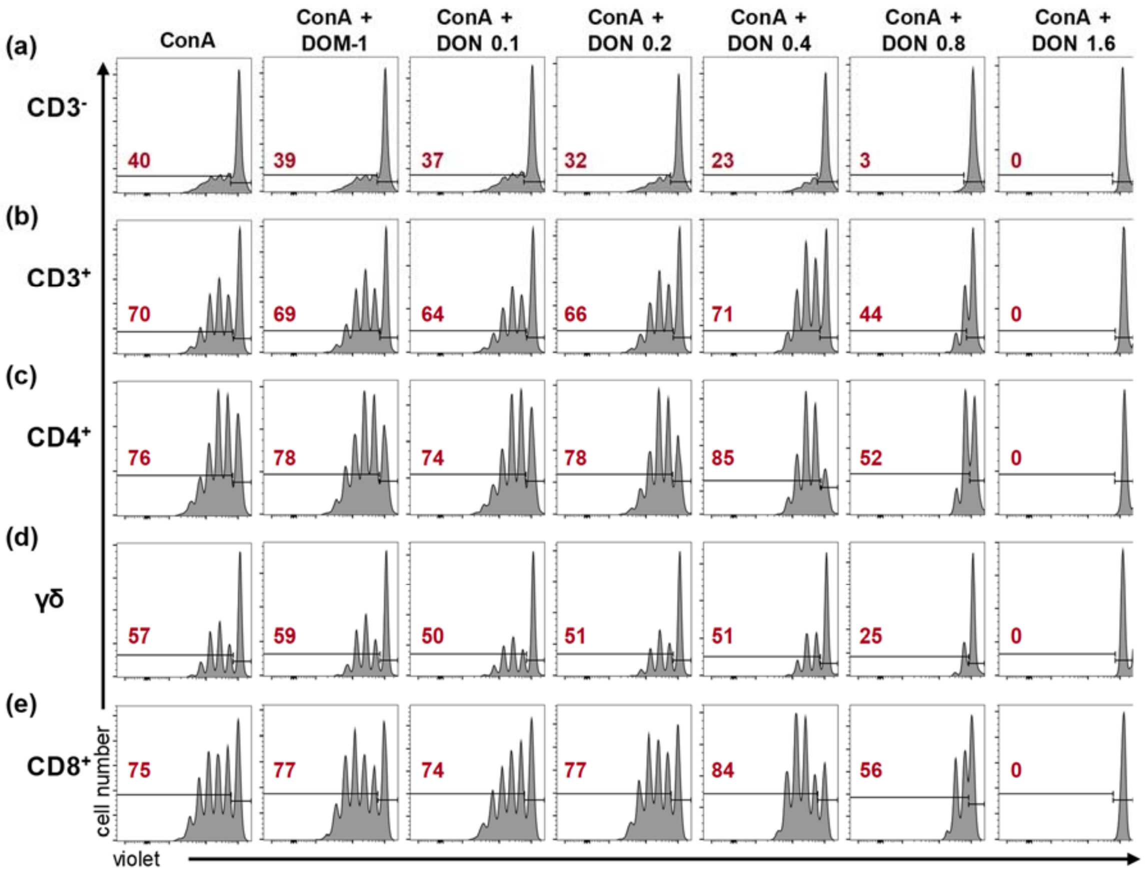

Figure S2. Proliferation of CD3[-] cells and major T-cell subsets in the presence of DON and DOM-1. After a violet proliferation staining and four days of in vitro cultivation in the presence of ConA, cells were harvested and labeled by Abs against CD3, CD4, CD8α and TCR-$\gamma\delta$ and analyzed for proliferation by FCM (**a**–**e**). Histograms show the fluorescence intensities of the violet proliferation dye in CD3[-] cells (**a**), CD3[+] T cells (**b**), CD4[+] T cells (**c**), $\gamma\delta$ T cells (**d**) and CD8[+] T cells (**e**) in the absence or presence of different DON concentrations (0.1, 0.2, 0.4, 0.8, 1.6 μM) and a single DOM-1 concentration (16 μM). The solid horizontal lines separate the parental (non-proliferating) population (on the right) and the proliferating generations (on the left). The numbers located on the left side in the histograms show the percentage of the proliferating cells. Representative data from one animal out of seven are shown.

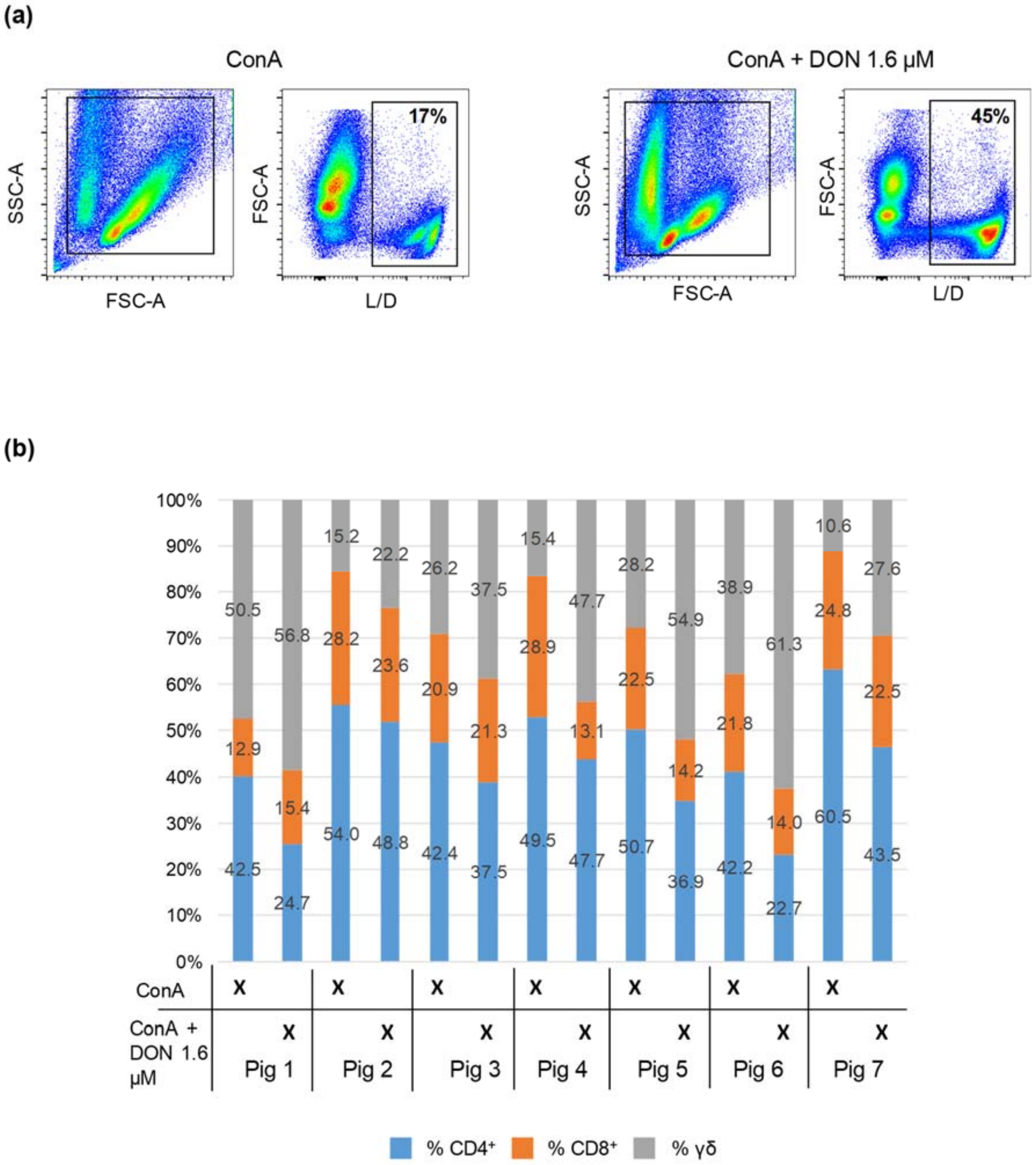

Figure S3. Relative distribution of CD4+, CD8+ and γδ T cells within dead CD3+ T cells. (**a**) After applying a large gate on FSC-A versus SSC-A to identify intact PBMCs, these cells were analyzed for fluorescence intensity of the live/dead (L/D) discrimination dye. Analyses were applied to cultures treated with ConA and ConA + 1.6 µM of DON. Cells with a high (L/D) signal were gated and subjected to the gating strategy depicted in Figure S1. Data of two representative samples from the same animal are shown. (**b**) Total CD3+ T cells were set to 100% (not shown) and the relative distribution of CD4+, CD8+ and γδ T cells were calculated for the different conditions for all investigated animals (n = 7). Data are displayed in stacked bar charts.

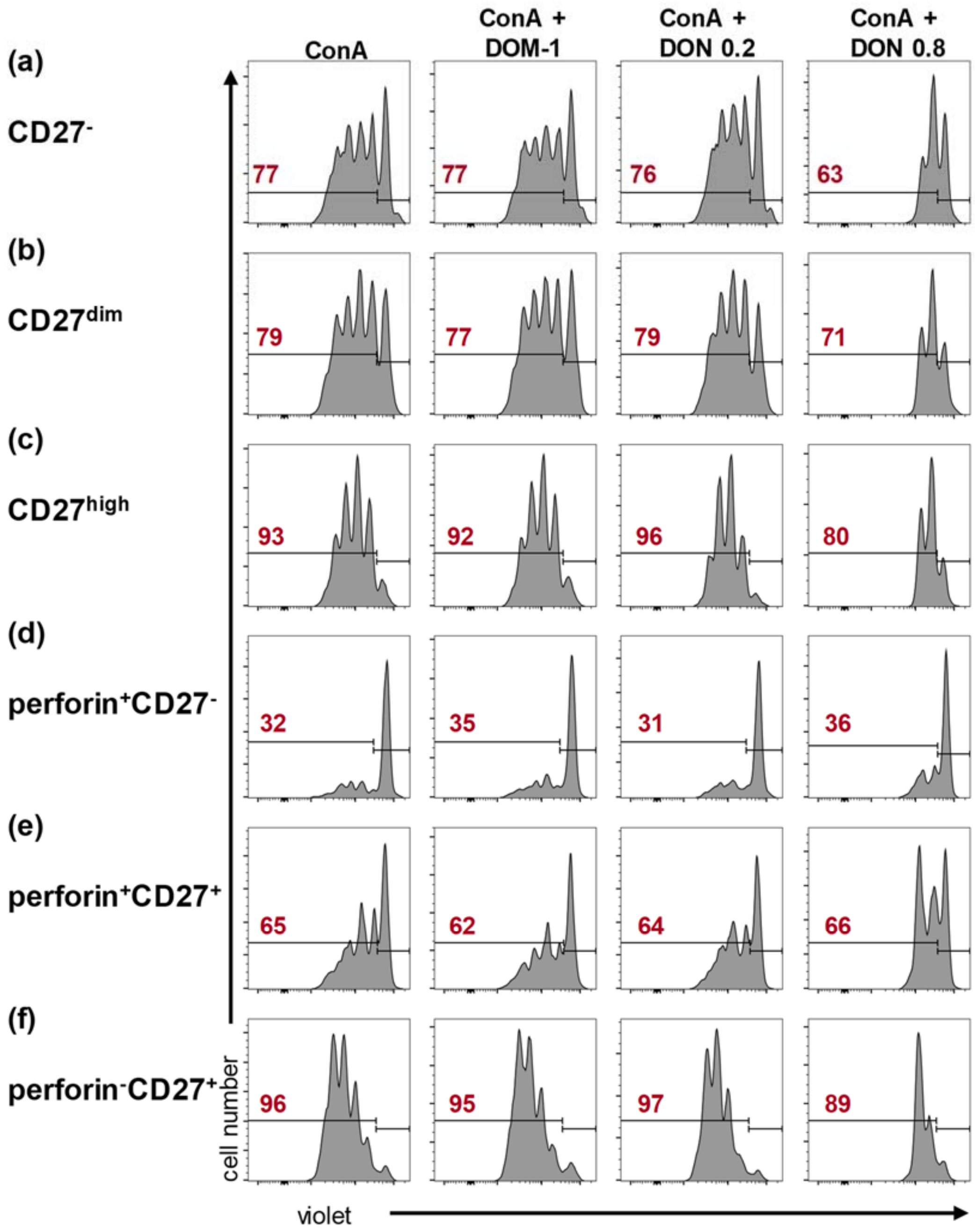

Figure S4. Proliferation of CD27-defined CD4⁺ and perforin/CD27-defined CD8⁺ T-cell subsets in the presence of DON and DOM-1. After violet proliferation staining and four days of in vitro cultivation in the presence of ConA alone or in combination with either two different DON concentrations (0.2 and 0.8 µM) or a single DOM-1 concentration (16 µM), cells were harvested and labeled by Abs against CD4, CD27, CD8α, CD8β and perforin and analyzed for proliferation by FCM (**a**–**f**). Histograms show proliferation of $CD27^-$ $CD4^+$ T cells (**a**), $CD27^{dim}$ $CD4^+$ T cells (**b**), $CD27^{high}$ $CD4^+$ T cells (**c**), $perforin^+CD27^-$ $CD8^+$ T cells (**d**), $perforin^+CD27^+$ $CD8^+$ (**e**) and $perforin^-CD27^+$ $CD8^+$ T cells (**f**) under the different cultivation conditions. The solid horizontal lines separate the parental (non-proliferating) population (on the right) and the proliferating generations (on the left). The numbers located on the left side in the histograms show the percentage of the proliferating cells. Representative data from one animal out of seven are shown.

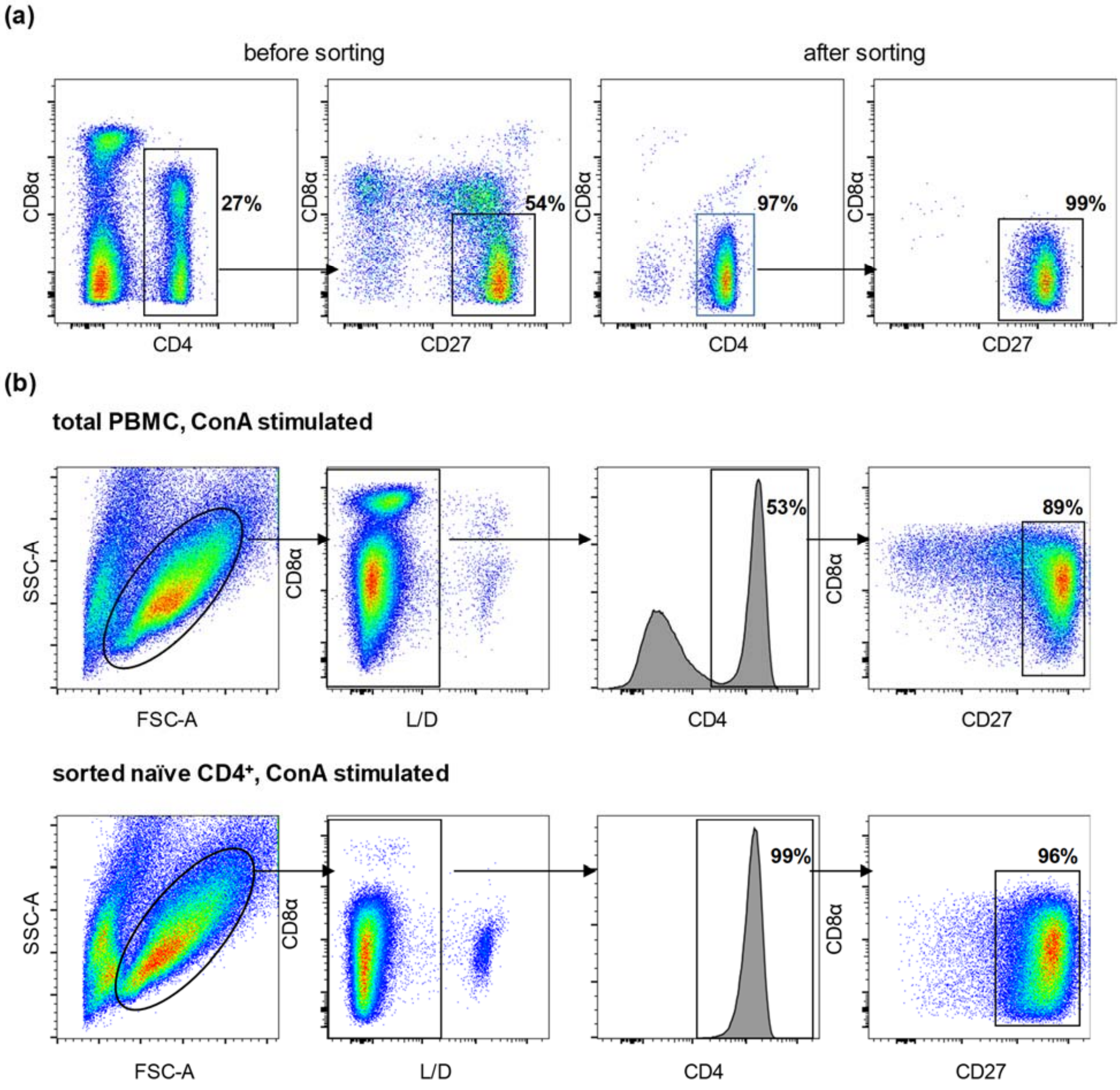

Figure S5. Gating strategy before and after sorting of naïve CD4+ T cells. PBMCs were thawed and stained for expression of the cell-surface markers CD4, CD8α, and CD27. (**a**) The two pseudocolor plots on the left indicate the gating strategy that was applied for the sorting CD8α-CD27high naïve CD4+ T cells. Within lymphocytes, CD4+ T cells were gated (pseudocolor plot on the left) and further analyzed for CD27 and CD8α expression. CD8α-CD27high CD4+ T cells were sorted by FACS. The two pseudocolor plots on the right part show the re-analysis for the purity of the cells after sorting. The same gating strategy as prior to the sorting was applied. (**b**) Gating strategy for violet stained non-sorted PBMCs (first row) and sorted naïve CD4+ T cells (second row) after four days of in vitro cultivation in the presence of ConA. After four days, cells were harvested and stained for expression of CD4, CD8α and CD27. Lymphocytes were gated according to their light scatter properties and dead cells were excluded by using a live/dead discrimination dye. Afterwards CD4+ T cells were gated (histograms) and further sub-gated for high expression levels of (CD27high) in both total PBMC (first row) and sorted CD8α-CD27high CD4+ T cells (second row). Data shown are representative for sorting experiments with PBMCs from three animals.

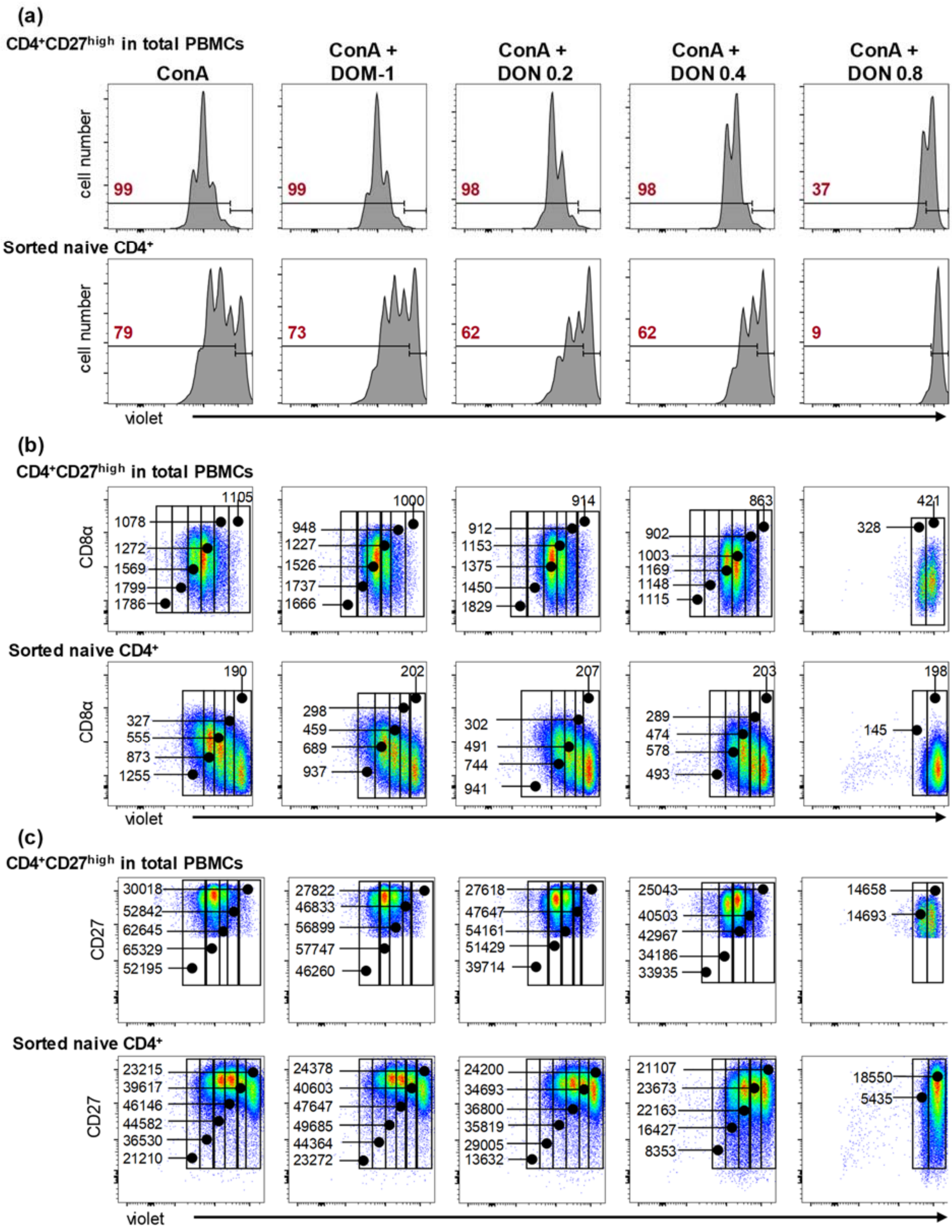

Figure S6. Influence of DON and DOM-1 on the expression of CD8α and CD27 in ConA stimulated sorted naïve CD4$^+$ T cells and CD4$^+$ T cells present in a PBMC bulk culture. Total PBMC or sorted CD8α^-CD27^{high} CD4$^+$ T cells were stained with violet proliferation dye and cultivated for four days in the presence of ConA alone or in combination with either three different DON concentrations (0.2, 0.4 and 0.8 μM) or a single DOM-1 concentration (16 μM). Four days later, cells were harvested and analysed for CD8α and CD27 expression as well as proliferation, as shown in the pseudocolor plots and histograms. Histograms (**a**) show proliferation of CD27^{high} CD4$^+$ T cells cultivated in bulk culture (first row) and of sorted CD27^{high} CD4$^+$ T cells (second row) under the different cultivation conditions.

The solid horizontal lines separate the parental (non-proliferating) population (on the right) and the proliferating generations (on the left). The numbers located on the left side in the histograms show the percentage of the proliferating cells. Following the gating strategy shown in Supplementary Figure 4, CD8α (**b**) and CD27 (**c**) expression were analyzed. The first rows of (**b**) and (**c**) show CD8α and CD27 expression, respectively, in CD27^{high} CD4^{+} T cells cultivated in PBMC bulk cultures (total PBMCs) and the second rows show their expression in FACS-sorted CD27^{high} CD4^{+} T cells. The cells gated on the right in each pseudocolor plot represent the parental (non-proliferating) population and the cells gated on the left are the proliferating cells in each generation. The numbers in the pseudocolor plots illustrate the median fluorescence intensity for CD8α (b) and CD27 (c) expression in each generation. Data shown are representative for sorting experiments with PBMCs from three animals.
